# Supplementary material for: Ecological and social constraints combine to promote evolution of non-breeding strategies in clownfish
Source: Commun Biol. 2020 Nov 6;3:649. doi: 10.1038/s42003-020-01380-8 (PMC7648053; doi:10.1038/s42003-020-01380-8)
Supplement: Supplementary file 2 — Reporting Summary [file 42003_2020_1380_MOESM2_ESM.pdf]

nature research

Corresponding author(s): Rebecca Branconi

Last updated by author(s): Sep 25, 2020

Reporting Summary

Nature Research wishes to improve the reproducibility of the work that we publish. This form provides structure for consistency and transparency in reporting. For further information on Nature Research policies, see our Editorial Policies and the Editorial Policy Checklist.

Statistics

For all statistical analyses, confirm that the following items are present in the figure legend, table legend, main text, or Methods section.

n/a	Confirmed	
☐	☒	The exact sample size (*n*) for each experimental group/condition, given as a discrete number and unit of measurement
☐	☒	A statement on whether measurements were taken from distinct samples or whether the same sample was measured repeatedly
☐	☒	The statistical test(s) used AND whether they are one- or two-sided<br>*Only common tests should be described solely by name; describe more complex techniques in the Methods section.*
☒	☐	A description of all covariates tested
☒	☐	A description of any assumptions or corrections, such as tests of normality and adjustment for multiple comparisons
☒	☐	A full description of the statistical parameters including central tendency (e.g. means) or other basic estimates (e.g. regression coefficient) AND variation (e.g. standard deviation) or associated estimates of uncertainty (e.g. confidence intervals)
☒	☐	For null hypothesis testing, the test statistic (e.g. *F*, *t*, *r*) with confidence intervals, effect sizes, degrees of freedom and *P* value noted<br>*Give P values as exact values whenever suitable.*
☒	☐	For Bayesian analysis, information on the choice of priors and Markov chain Monte Carlo settings
☒	☐	For hierarchical and complex designs, identification of the appropriate level for tests and full reporting of outcomes
☒	☐	Estimates of effect sizes (e.g. Cohen's *d*, Pearson's *r*), indicating how they were calculated

Our web collection on statistics for biologists contains articles on many of the points above.

Software and code

Policy information about availability of computer code

Data collection	No software was used to collect the data; All the data were collected in the field.
Data analysis	All analyses were done in R v. 3.4.2 ‘Short Summer’ using Fisher’s exact tests for contingency tables.

For manuscripts utilizing custom algorithms or software that are central to the research but not yet described in published literature, software must be made available to editors and reviewers. We strongly encourage code deposition in a community repository (e.g. GitHub). See the Nature Research guidelines for submitting code & software for further information.

Data

Policy information about availability of data

All manuscripts must include a data availability statement. This statement should provide the following information, where applicable:

- Accession codes, unique identifiers, or web links for publicly available datasets
- A list of figures that have associated raw data
- A description of any restrictions on data availability

We will provide a full data availability statement and a full code availability in the manuscript

Field-specific reporting

Ecological, evolutionary & environmental sciences study design

All studies must disclose on these points even when the disclosure is negative.

Study description	ECOLOGICAL CONSTRAINTS EXPERIMENT #1:To test the hypothesis that clownfish non-breeding Individuals do not disperse to breed elsewhere because of strong ecological constraints in the form of risk of mortality during dispersal, we experimentally tested the critical prediction that non-breeding individuals will disperse when the risk of moving between anemones is reduced. Risk was to be manipulated by presenting alternative anemones in succession at a distance of 0.5 m and 5.0 m from focal groups. To explore the effect of variation in the alternative option, two classes of anemones were used: empty anemones or anemones with a breeding male. We left alternative anemones alongside the home anemones for two days, to allow the focal non-breeder sufficient time to make a choice. The morning of the third day, we recorded whether focal non-breeders (rank 3) had moved to the alternative anemone.<br><br>ECOLOGICAL CONSTRAINTS EXPERIMENT #2:To test the critical prediction that clownfish non-breeders will not return to their home anemone when the risk of moving between anemones is increased, once more we presented alternative anemones at a distance of 0.5 m and 5.0 from focal groups. As in the first experiment, two classes of anemones were used at each distance: empty anemones or anemones with a breeding male. In this case, however, we relocated the focal non-breeder from the home anemone to the alternative .We left this set-up for two days, to allow the focal non-breeder sufficient time to make a choice. The morning of the third day, we recorded whether the focal non-breeder had returned to its home anemone.<br><br>SOCIAL CONSTRAINTS EXPERIMENT:To test the hypothesis that clownfish non-breeding Individuals do not contest for breeding positions because of strong social constraints in the form of evictions of non-cooperative individuals, we experimentally tested the critical prediction that non-breeding Individuals will contest for breeding positions when the probability of winning a contest is increased. To test this prediction, we used 16 focal groups. All individuals in each focal group were caught and measured to the nearest 0.1 mm using calipers, and the largest non-breeder (rank 3) was removed. Then, we introduced two types of rank 3 individuals to the focal group: a non-breeder less than the 80% of the size of the breeding male (rank 3') or a non-breeder more than 80% of the size of the breeding male (rank 3''). Each focal group received rank 3' and rank 3'', one at a time, on different days, in random order. We left introductions overnight and, the following day, we noted whether there had been a contest, as indicated by an eviction. If introducees disappeared overnight, they were considered evicted because i) they do not leave voluntarily and ii) mortality without eviction is rare. If introducees were still present, they were considered evicted if they spent more than 3 minutes out of 5 outside the anemone (i.e. with their full body length outside of the range of anemone tentacles) and considered tolerated if they were still present and spent less than 3 minutes outside of the anemone.
Research sample	We located 186 groups of clown anemonefish (Amphiprion percula) and all the fish within each group were measured.<br>For the ECOLOGICAL CONSTRAINTS EXPERIMENT #1 and #2: We used 32 anemones and relative groups as focal groups; 6 anemones were used as alternative anemones (3 were empty and 3 with a breeding male)<br>For the SOCIAL CONSTRAINTS EXPERIMENT: We used 16 anemones and relative groups as focal groups and 32 non breeders from other 32 different groups.
Sampling strategy	Each group in each experiment was tested twice so that each group serverd as internal control.
Data collection	All work was conducted using SCUBA at depths up to 20 meters. Data collection (notes on individuals dispersion and presence/ absence) was performed by Rebecca Branconi and Tina Barbasch
Timing and spatial scale	The experiments were conducted from June to September 2018 across 12 different inshore reefs.<br>186 groups of A.percula were captured and measured in June 2018. During the whole month of June, several trials were performed to optimize the methods and the procedures to be used for the 3 experiments. The collection of the data started on the 5th of July and ended on the 3rd of September.<br>For the ECOLOGICAL CONSTRAINTS EXPERIMENT #1 and #2, the collection of each data point required 6 days.<br>For the SOCIAL CONSTRAINTS EXPERIMENT, the collection of each data point required 4 days.
Data exclusions	No data were exlcuded from the analyses
Reproducibility	To ensure the reproducibility of our experimental design, during the whole month of June, several trials were performed to optimize the methods and the procedures to be used for the 3 experiments presented in this study.
Randomization	The order for the introductions was randomized.
Blinding	Because of the nature of these underwater field experiments it was impossible to conduct them blinded

Did the study involve field work? ☒ Yes ☐ No

Field work, collection and transport

Field conditions	Depths between 0-20 meters; temperatures between 80-84 degree fahrenheit
Location	12 inshore reefs near Mahonia Na Dari Research and Conservation Centre, Kimbe bay, Papua New Guinea
Access & import/export	All work was performed with the approval of the Institutional Animal Care and Use Committee, Boston University (Protocol number: 17/001) and the Government of Papua New Guinea.

Disturbance	We captured all fish using hand nets, placed them inside clear plastic bags, laid them against a slate, and measured their standard length to the nearest 0.1 mm using calipers. This entire procedure was conducted underwater to minimize disturbance and return all individuals to their anemone within a few minutes.<br>When possible, the transport of the fish and of the alternative anemones was conducted underwater using SCUBA to minimize the use of a boat.

Reporting for specific materials, systems and methods

We require information from authors about some types of materials, experimental systems and methods used in many studies. Here, indicate whether each material, system or method listed is relevant to your study. If you are not sure if a list item applies to your research, read the appropriate section before selecting a response.

Materials & experimental systems

n/a	Involved in the study
☒	☐ Antibodies
☒	☐ Eukaryotic cell lines
☒	☐ Palaeontology and archaeology
☐	☒ Animals and other organisms
☒	☐ Human research participants
☒	☐ Clinical data
☒	☐ Dual use research of concern

Methods

n/a	Involved in the study
☒	☐ ChIP-seq
☒	☐ Flow cytometry
☒	☐ MRI-based neuroimaging

Animals and other organisms

Policy information about studies involving animals; ARRIVE guidelines recommended for reporting animal research

Laboratory animals	n/a
Wild animals	We located 186 magnificent sea anemones (Heteractis magnifica). Each anemone was occupied by a single group of clown anemonefish (Amphiprion percula). Groups consisted of a breeding pair and zero to three non-breeders. Individuals were identified based on natural variation in their colour markings.<br>The anemones that were movable e.g., attached to small rocks or only loosely attached to the hard substrate, were collected and used as alternative anemones for the ecological constraints experiments.<br>During the experiment, unused fish from the alternative anemones were kept in the laboratory at Mahonia Na Dari Research and Conservation Center. At the end of the experiment, all fish and anemones were returned to their original location
Field-collected samples	n/a
Ethics oversight	All work was performed with the approval of the Institutional Animal Care and Use Committee, Boston University (Protocol number: 17/001) and the Government of Papua New Guinea.

Note that full information on the approval of the study protocol must also be provided in the manuscript.
